# DenseNet weed recognition model combining local variance preprocessing and attention mechanism

**DOI:** 10.3389/fpls.2022.1041510

**Published:** 2023-01-12

**Authors:** Ye Mu, Ruiwen Ni, Lili Fu, Tianye Luo, Ruilong Feng, Ji Li, Haohong Pan, Yingkai Wang, Yu Sun, He Gong, Ying Guo, Tianli Hu, Yu Bao, Shijun Li

**Affiliations:** ^1^ College of Information Technology, Jilin Agricultural University, Changchun, China; ^2^ Jilin Province Agricultural Internet of Things Technology Collaborative Innovation Center, Jilin Agricultural University, Changchun, Jilin, China; ^3^ Jilin Province Intelligent Environmental Engineering Research Center, Jilin Agricultural University, Changchun, Jilin, China; ^4^ Jilin Province Information Technology and Intelligent Agriculture Engineering Research Center, Jilin Agricultural University, Changchun, Jilin, China; ^5^ Faculty of Agronomy, Jilin Agricultural University, Changchun, China; ^6^ School of Management, Changchun University, Changchun, China; ^7^ College of Information Technology, Wuzhou University, Wuzhou, China; ^8^ Guangxi Key Laboratory of Machine Vision and Intelligent Control, Wuzhou University, Wuzhou, Guangxi, China

**Keywords:** weed recognition, DenseNet, attention mechanism, image preprocessing, local variance

## Abstract

**Introduction:**

The purpose of this paper is to effectively and accurately identify weed species in crop fields in complex environments. There are many kinds of weeds in the detection area, which are densely distributed.

**Methods:**

The paper proposes the use of local variance pre-processing method for background segmentation and data enhancement, which effectively removes the complex background and redundant information from the data, and prevents the experiment from overfitting, which can improve the accuracy rate significantly. Then, based on the optimization improvement of DenseNet network, Efficient Channel Attention (ECA) mechanism is introduced after the convolutional layer to increase the weight of important features, strengthen the weed features and suppress the background features.

**Results:**

Using the processed images to train the model, the accuracy rate reaches 97.98%, which is a great improvement, and the comprehensive performance is higher than that of DenseNet, VGGNet-16, VGGNet-19, ResNet-50, DANet, DNANet, and U-Net models.

**Discussion:**

The experimental data show that the model and method we designed are well suited to solve the problem of accurate identification of crop and weed species in complex environments, laying a solid technical foundation for the development of intelligent weeding robots.

## 1 Introduction

With the development of modern agricultural technology, China’s grain production has been increasing year by year, but there are still some problems that cannot be ignored. Among them, weeds are one of the main hazards that affect crop yield and quality (Li et al., 2013; Li et al., 2013). Weeds not only compete with crop seedlings for fertilizer, light, water, and growing space, causing crop failure, but also contribute to the occurrence and spread of pests and diseases that threaten crop survival (Nardecchia et al., 2021). At present, the main weeding method is manual operation, and farmers often use large-area random spraying of herbicides in the weeding process, which can cause great environmental pollution and chemical residues and also produce great harm to people’s health. Moreover, large-scale weeding operations are not targeted, weeding efficiency is often not high, and repeated weeding is required (Patches et al., 2017; Li, 2018; Duan et al., 2019). With the introduction of smart agriculture, the implementation of precision spraying can effectively control the growth of weeds in the field and maximize the utilization of pesticides and reduce drug residues. The accurate identification of weeds can lay the theoretical foundation and provide technical support for the implementation of precision spraying. In recent years, how to solve the problem of improving the efficiency of field operation and solving the shortage of agricultural labor and enhancing the accuracy of automatic weeding has become the main research content at present (Li, 2018; Duan et al., 2019), in which the automatic weed identification technology based on machine vision and image processing is the research hotspot (Yan, 2018; Yuan et al., 2020).

Traditional image processing methods usually use wavelet analysis, Bayesian discriminant models, and support vector machines (SVMs) to achieve crop and weed recognition based on features such as weed color, shape, texture, and spatial distribution and combinations of these features (Wang and Li, 2016; Zheng et al., 2017; Elstone et al., 2020; Hou et al., 2020). Although these methods are less difficult to detect, the environment of the general crop growing area is complex, and the robustness of the methods using weed-specific features for identification is poor and the accuracy of identification is not high (Jordan and Mitchell, 2015; Chen and Wang, 2017; Rojas et al., 2017; Bakhshipour et al., 2017).

With the development of computer technology, fast and accurate machine vision recognition technology is more and more widely used in weed recognition. Many scholars have carried out relevant research. In terms of weed identification, Dos et al. (2017) compared AlexNet with SVM and random forest model and concluded that AlexNet architecture can better identify soybean, soil, and broad-leaved weeds than other models. Potena et al. (2016) proposed a multistep vision system based on RGB+NIR (near infrared) images, using two different convolutional neural network (CNN) architectures to classify crops and weeds; Jiang et al. (2020) used the graph convolution neural network to identify three types of crops and weeds on the AlexNet, VGG-16, and ResNet-101 network models, and the average recognition accuracy of ResNet-101 reached 96.51%. Peng et al. (2020) took weeds in a rice field as the research object. During the training of the deep convolution neural network, the optimizer under a random gradient was used to optimize the parameters. Among them, the VGG-16-SGD model had the highest accuracy, and its average *F* (*F*-measure) value was 0.977. Deng et al. (2021) used the pretrained CNN model combined with the migration learning method to identify the weeds in the field of rice seedlings. Among them, the correct recognition rate of the VGG-16 model reached 97.8%. Zhang Xinming and others proposed a recognition method for corn and weeds based on the improved probabilistic neural network (PNN) and used the suboptimal search method to select the most effective features to construct the feature vector, which improved the recognition performance and speed. It can be seen from the above literature that the weed recognition method based on deep learning can well solve the problem of extracting specific features in traditional image processing, and the accuracy is also improved to a certain extent. However, there are still problems such as the following: 1) In the crop field under a complex environment, when the environment around weeds changes, the existing deep learning model has the problem of weak generalization ability for weed recognition. 2) In the process of feature extraction, the convolutional neural network extracts a large amount of invalid background information because of the diversity of background and the large proportion of image pixels, which affects the recognition results and cannot maintain a high recognition accuracy.

To address the above problems, this paper proposes a weed recognition model based on an improved dense convolutional network (DenseNet) (Huang et al., 2017) to improve the recognition accuracy and the generalization ability of the network by introducing an efficient channel attention (ECA) mechanism (Wang et al., 2020) and a local variance algorithm (Zhao et al., 2019) to suppress the extraction of invalid background features while enhancing weed feature extraction, thus improving the recognition accuracy and the generalization ability of the network to ensure efficient and accurate weed recognition in complex environments.

**Figure 1:**
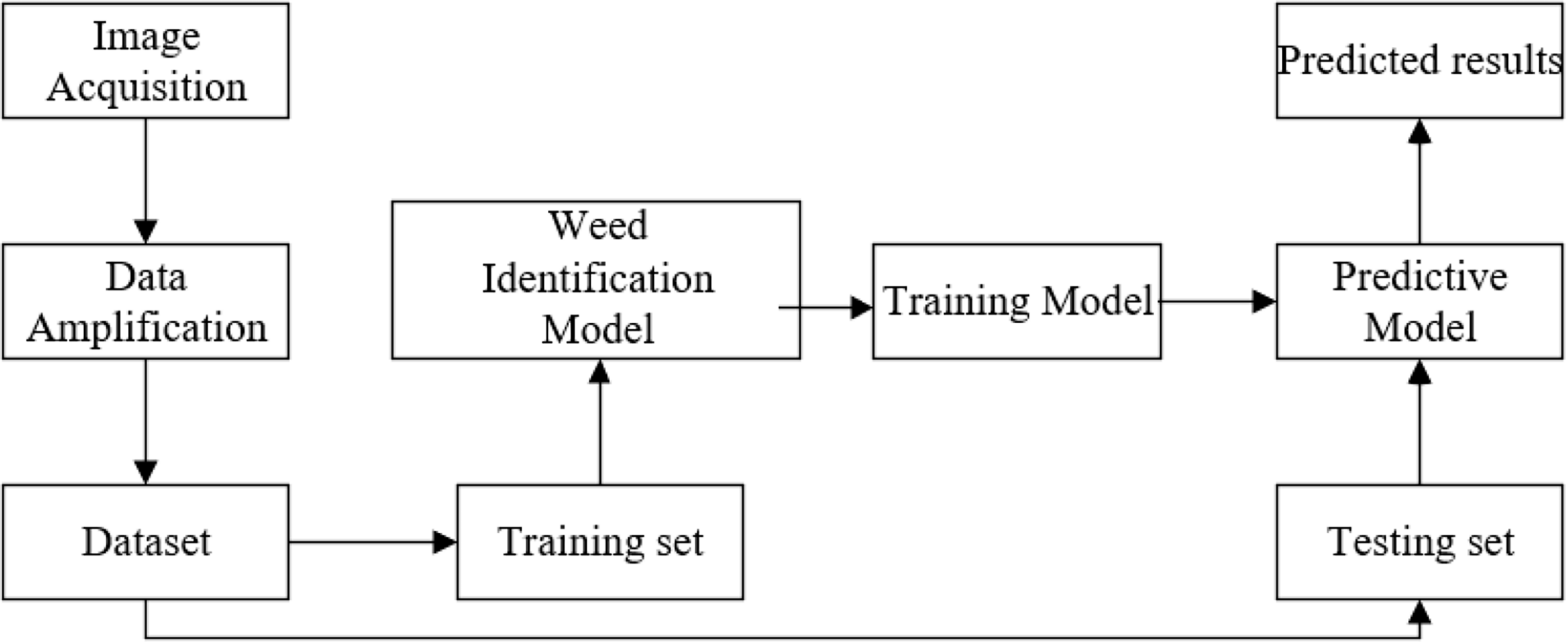
The weed identification process.

In this paper, weed identification in the field is performed by improving DenseNet. The steps are shown in **Figure 1**. First, we collected crop and weed images. The weed dataset is built by amplifying the data to ensure diversity of the data. Secondly, the training set is input to the weed recognition model, and then the trained weights were loaded into the model to get the prediction model. Finally, we input the test set to get the prediction results.

## 2 Data processing

### 2.1 Dataset

**Figure 2:**
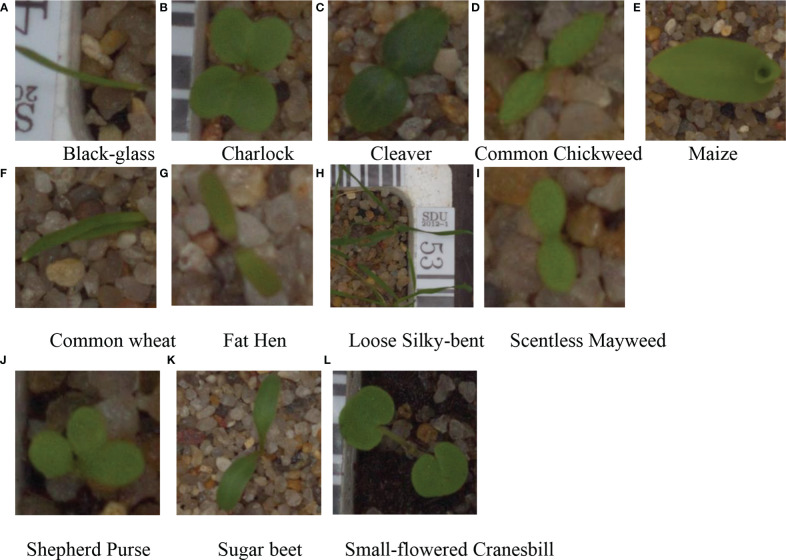
Partial dataset. **(A)** Black-glass **(B)** Charlock **(C)** Cleaver **(D)** Common Chickweed **(E)** Maize **(F)** Common wheat **(G)** Fat Hen **(H)** Loose Silky-bent **(I)** Scentless Mayweed **(J)** Shepherd Purse **(K)** Sugar beet **(L)** Small-flowered Cranesbill.

Due to the complex ground conditions in the field, we used the segmentation model from a published paper (Mu, 2022) to segment the captured field data into a single image of only one plant, and to show that our model can distinguish crops from weeds, we chose corn seedlings because they are more similar to weeds. Because we do not have enough data, we choose the public dataset to train the model. This dataset (Giselsson et al., 2017) mainly uses images of crops and weed seedlings provided by the Computer Vision and Signal Processing Group of the Department of Engineering, Aarhus University, Denmark. The dataset is divided into 12 categories with 5,539 images, mainly black-grass, charlock, cleavers, common chickweed, common wheat, fat hen, loose silky-bent, maize, scentless mayweed, shepherd purse, small-flowered cranesbill, and sugar beet. The selection of the dataset was convenient to demonstrate that our experiment can effectively distinguish between maize seedlings and weeds. In the actual training of the model, we chose to treat the original dataset as follows (considering the size and number of datasets):

1) In order to prevent overfitting due to the limited number of images, this paper uses data augmentation techniques in deep learning to geometrically transform the existing dataset, increase the diversity of data by expanding the number of corn and weed images, avoid the appearance of the network learning irrelevant features, and then learn more features related to the data to improve the recognition ability of the model. In this paper, the collected weed and corn images are expanded to twice the original dataset by using two data augmentation methods: adding noise and random directional flipping, resulting in a total of 11,078 images. Among them, 8,862 images are in the training set and 2,216 images in the test set.

2) To meet the input requirements of the network for image pixels, the image pixels are first adjusted to 256 × 256 during training and then cropped from the center to obtain a 224 × 224 pixel image, and the cropped part of the weed dataset image is shown in **Figure 2**.

### 2.2 Data processing

The main objects targeted in this paper are weed images with complex backgrounds taken in a natural lighting environment, where the light intensity and background of the images are different. Background segmentation of the original dataset is performed to improve the accuracy of the weed recognition model in complex natural backgrounds, to extract the weed parts of interest in the images, and to remove the background parts that are not useful for image recognition. Through the analysis of the dataset, it was found that the color characteristics of both weeds and seedling corn were green, which differed significantly from the color of the background such as soil. Therefore, in this paper, we choose the super green algorithm (2G-R-B) (Wang and Yang, 2018) by normalization, which can increase the weight of the green channel in the RGB image and thus suppress the non-green background part of the image. Using this feature can quickly and effectively separate the weedy regions in the natural background, and the specific procedure of the super green algorithm is as follows (Formulae 1, 2):


$$\{\begin{array}{c} r=\frac{R}{R+G+B}\\ g=\frac{G}{R+G+B}\\ b=\frac{B}{R+G+B} \end{array}\;\left(1\right)$$

$$ExG=\{\begin{array}{c} 2g-r-b,2g\geq r+b\\ 0,2g<r+b \end{array}\;\left(2\right)$$

In the formula, *R*, *G*, and *B* are the pixel channel values in the RGB color space, and *ExG* is the super green image.

After obtaining the grayscale image by the above method, this paper chooses to perform a secondary processing of the resulting grayscale image by local variance preprocessing. The local variance can be used to measure the sharpness of grayscale variation in the volume region. For a pixel (*x*, *y*), *f*(*x*, *y*) is its gray value. Centered on this point, select 3 × 3 as the calculation neighborhood of local variance, and the local variance *v*(*x*, *y*) of this point is expressed as (Formula 3):


$$v\left(x,y\right)=\frac{\sum_{i=-1}^{1}\sum_{j=-1}^{1}{\left[f\left(x-i,y-j\right)-\bar{f\left(x,y\right)}\right]}^{2}}{9}\;\left(3\right)$$

where *f*(*x*, *y*) is the grayscale value of pixel point (*x*, *y*), which is the mean value of the grayscale value of the 9 pixel points in the window, and its expression is shown in Formula 4.


$$\bar{f\left(x,y\right)}=\frac{\sum_{i=-1}^{1}\sum_{j=-1}^{1}f\left(x-i,y-i\right)}{9}\;\left(4\right)$$

**Figure 3:**
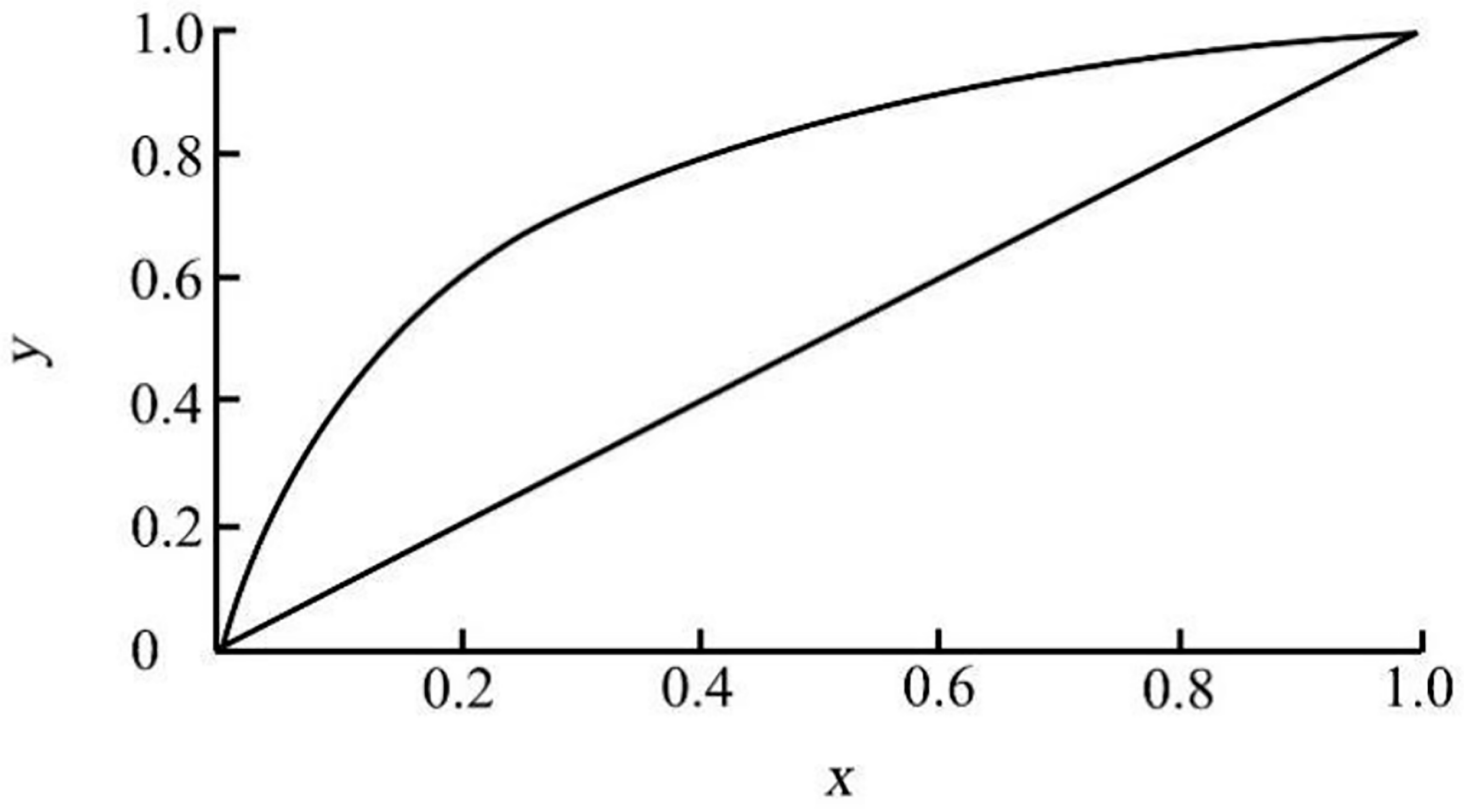
Comparison of two normalization functions.


Since both maize seedlings and weeds are green, selecting the *g* component for further image processing can try to maintain the information integrity of the image. In order to facilitate the function processing of image data, the image data are unified into double type, and the gray range of the image is [0,1]. Therefore, the variation range of *F*(*x*, *y*) is [0,1]. Substituting it into Formula (3), the difference between *F*(*x*, *y*) is compressed after square operation, resulting in the difference of variance data not obvious enough. Therefore, the calculated variance needs to be normalized. When lawn grass is sparse, the background gray value of lawn grass can be suppressed to a certain extent after linear normalized local variance operation. However, in some areas with dense turfgrass, the gray difference between turfgrass and weeds is still not significant after linear normalized local variance calculation, which cannot effectively distinguish the lawn background and weed prospect. So, we use non-linear normalization. In the places with sparse and dense grass leaves, the local variance can have a good inhibitory effect on the lawn background and retain the preliminary enhancement effect on weeds. The non-linear normalization formula adopted is shown in Formula 5, and its comparison output with linear normalization is shown in **Figure 3**.

$$V(x,y)=\frac{a\cdot v\left(x,y\right)}{b\cdot v\left(x,y\right)+\left(a-b\right)}\;\left(5\right)$$

Where *V*(*x*, *y*) is the normalized variance and *a*, *b* are the non-linear normalization coefficients. In this study, *a* = 6 and *b* = 5 (**Figure 3**).


$$g\left(x,y\right)=\frac{f\left(x,y\right)}{\exp\left[kV^{2}\left(x,y\right)\right]-m}\;(6)$$

Bring the normalized local variance into Formula 6 to obtain the preprocessed image *g*(*x*, *y*).

**Figure 4:**
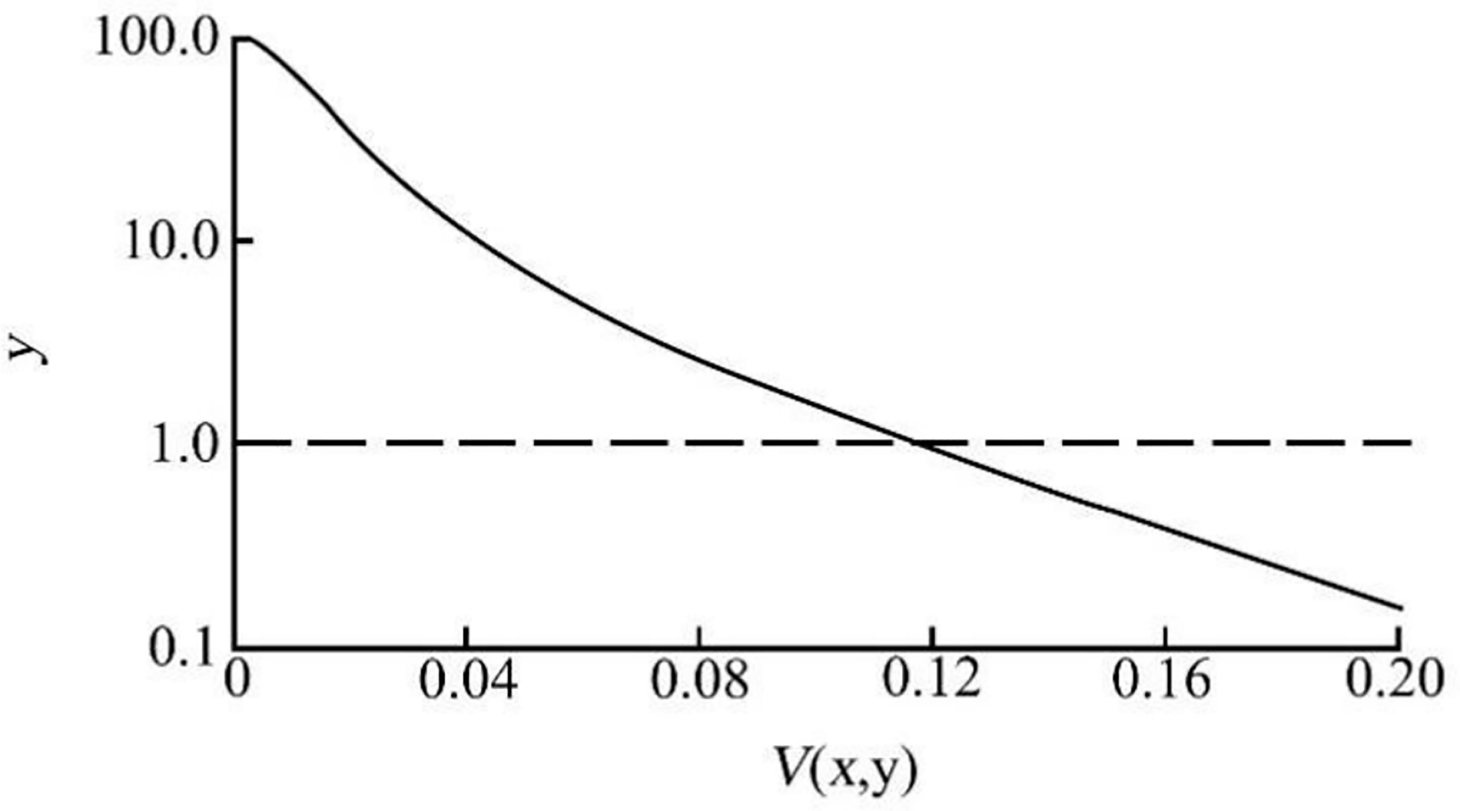
Preprocessing function output. Note: $—y=\frac{1}{\exp\left[kV^{2}\left(x,y\right)\right]-m};--y=1$ .

Where *k* and *m* are the optimization coefficients, and *V*_2_(*x*, *y*) is the square of normalized local variance *V*(*x*, *y*) at pixel point (*x*, *y*). If *k* > 0 is satisfied, the greater the *k* is, the more obvious the gray suppression effect is at the place with small local variance in the preprocessed image. *M* satisfies 0 < *m* < 1 to adjust the gain effect at small variance. In this study, *k* = 50 and *m* = 0.99 are selected, and the output results of the preprocessing function are shown in **Figure 4**.

As can be seen from **Figure 4**, when the normalized local variance is less than 0.12, the gray value of the corresponding part is enhanced. When the normalized local variance is greater than 0.12, the output value decreases rapidly below 1.0, and the corresponding gray value is suppressed. The preprocessed image is shown in **Figure 4**. The average gray value of the visible background is greatly reduced, and the gray value of the foreground is more prominent. However, there are still a considerable number of strip or point areas in the background area, and the gray value is similar to the foreground, which has a certain impact on image segmentation. Therefore, it is necessary to introduce an enhancement algorithm to expand the gray difference between the foreground and the background and suppress the residual noise in the background area.

**Figure 5:**
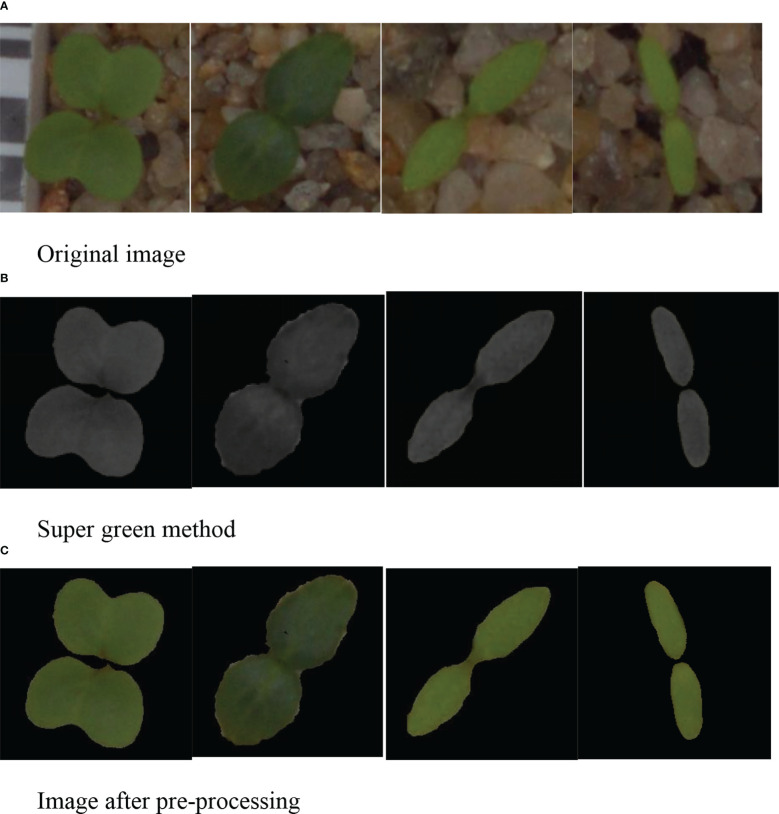
Image preprocessing. **(A)** Original image **(B)** Super green method **(C)** Image after preprocessing.

Finally, through the open and close operations of image morphology, the noise filtering and hole filling are realized. The image mask RGB original image and the processed binary image are used for the “and” operation, and the segmented image is shown in **Figure 5**.

**Figure 6:**
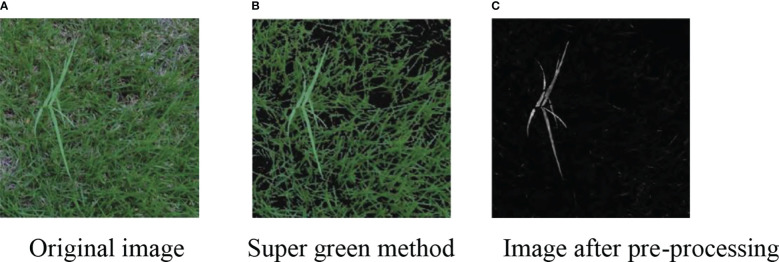
Segmentation of the complex background image. **(A)** Original image **(B)** Super green method **(C)** Image after pre-processing.

Since the difference between the weed background in **Figure 6** and the weed itself is large, it does not well reflect the superiority of our designed image preprocessing method, so we demonstrate the superiority of our designed method by shooting part of the weed image. The weed background in the captured weed image is highly similar to the color of the weed itself, and the segmentation process is more complicated. However, we still choose the dataset of Giselsson et al. (2017) due to the small amount of data.

## 3 Model building

### 3.1 Attention mechanism

**Figure 7:**
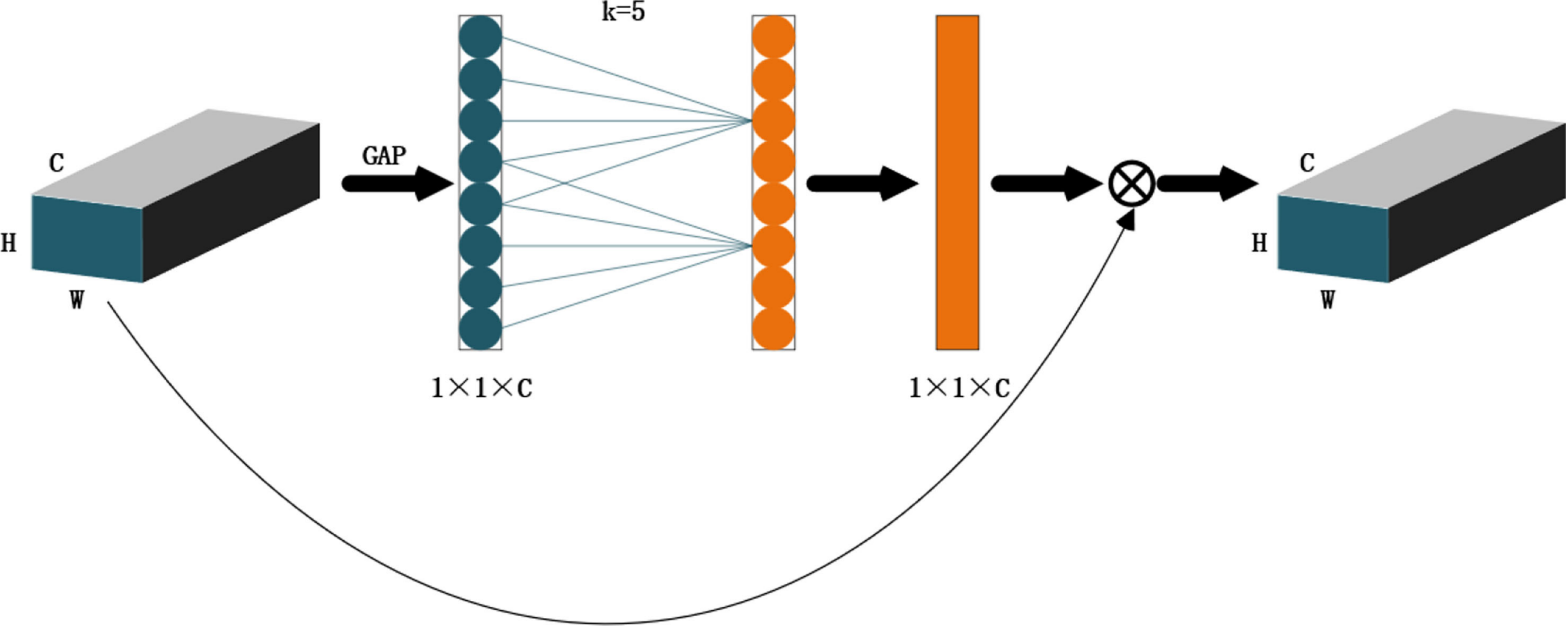
The ECA module structure diagram. Note: C is the number of channels, H is the height of the input data, W is the width of the input data, and K is the convolution local interaction size. The global average pooling of gap, σ, activates the function for sigmoid.

In the corn field under a complex environment, weeds and corn grow together, and the background is diverse. By adding attention mechanism, the weed features in the image are extracted. This paper adopts a lightweight attention module ECA net to improve the performance of the deep convolution neural network. By using an efficient attention module to combine the depth of the feature map with spatial information, focus on the extraction of important features, and inhibit the extraction of non-important features, we can effectively improve the recognition accuracy of field weeds in a complex environment. **Figure 7** shows the structure diagram of the ECA net. Firstly, the input characteristic map is globally averaged and pooled and a single value is used to represent the characteristic layer of each channel. Secondly, the one-dimensional convolution with the size of *K* is used to generate weights for each channel to obtain the interdependence between each channel. Sigmoid activation function is added for normalization. Finally, the weights of the generated channels are weighted to the input feature map by multiplication to strengthen the extraction of important features.

ECA uses one-dimensional convolution cross-channel interaction with size to replace the full connection layer, which can effectively reduce the amount of calculation and complexity of the full connection layer and then generate weights for each channel (Formula 7):


$$\omega =\delta (CID_{k}\left(y\right))\left(7\right)$$

Where *ω* is the channel weight, *δ* is the sigmoid activation function, and *CID* is the one-dimensional convolution. The more channels of the input characteristic graph, the greater the value of the local interaction, so the value of *K* is directly proportional to the number of channels *C*. In this paper, the *K* value is adaptively determined by the function related to the channel dimension (Formula 8):


$$C=2^{(\gamma \cdot k-b)}\;\left(8\right)$$

To sum up, it can be concluded that (Formula 9):


$$k={\left|\frac{{\text{log}}_{2}(C)}{\gamma }+\frac{b}{\gamma }\right|}_{\text{odd}}\;\left(9\right)$$

### 3.2 Model improvement

In the deep learning model in recent years, the CNN has always been absolutely dominant. ResNet, GoogLeNet, VGG, and other excellent networks are built based on the CNN. However, deep CNN has always had a problem: data are likely to gradually disappear after multilayer propagation. ResNet promotes the flow of data between layers to a certain extent through the “skip connection” structure. However, the network layer close to the output still does not fully obtain the characteristic diagram in front of the network.

**Figure 8:**
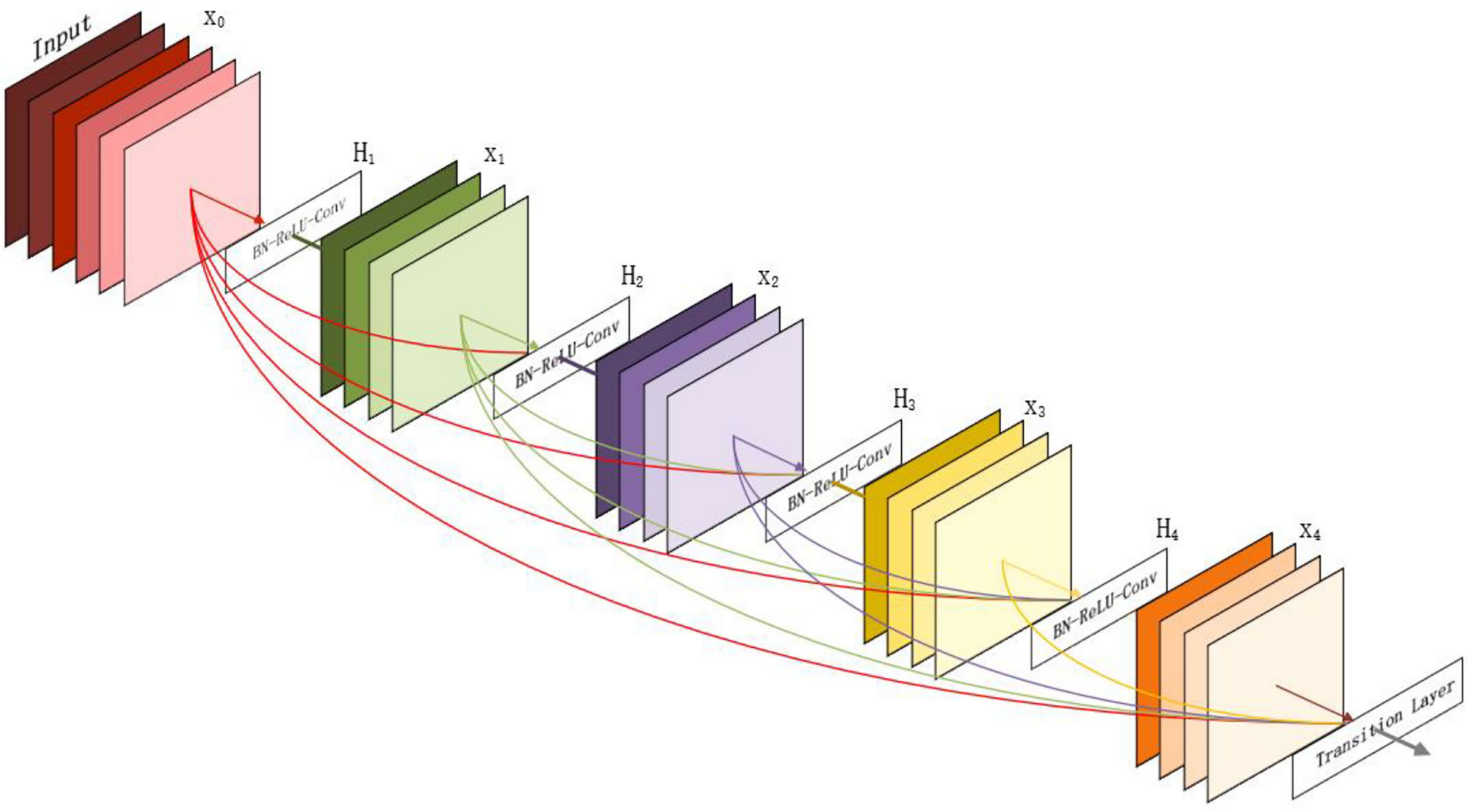
The DenseNet model diagram.

In the CVPR2017 best paper densely connected revolutionary networks, the author proposes a new DenseNet network (**Figure 8**). The starting point is to solve the redundancy problem of ResNet. Fewer parameters are used, which also alleviates the problem of gradient disappearance, and the network is easier to train. The difference between DenseNet and ResNet in mathematical expression is that the skip layer addition in ResNet is changed into concatenate connection operation. However, the color and appearance of weeds and maize seedlings are similar, so it is necessary to extract plant feature points more intensively. U-Net (Ronneberger et al., 2015) is a network structure with complete symmetry of convolutional coding and convolutional decoding. It can capture different levels of features and integrate them through feature superposition. Different levels of features, or receptive fields of different sizes, have different sensitivities to target objects of different sizes. Therefore, we choose to combine U-Net and DenseNet to form a new network structure.

**Figure 9:**
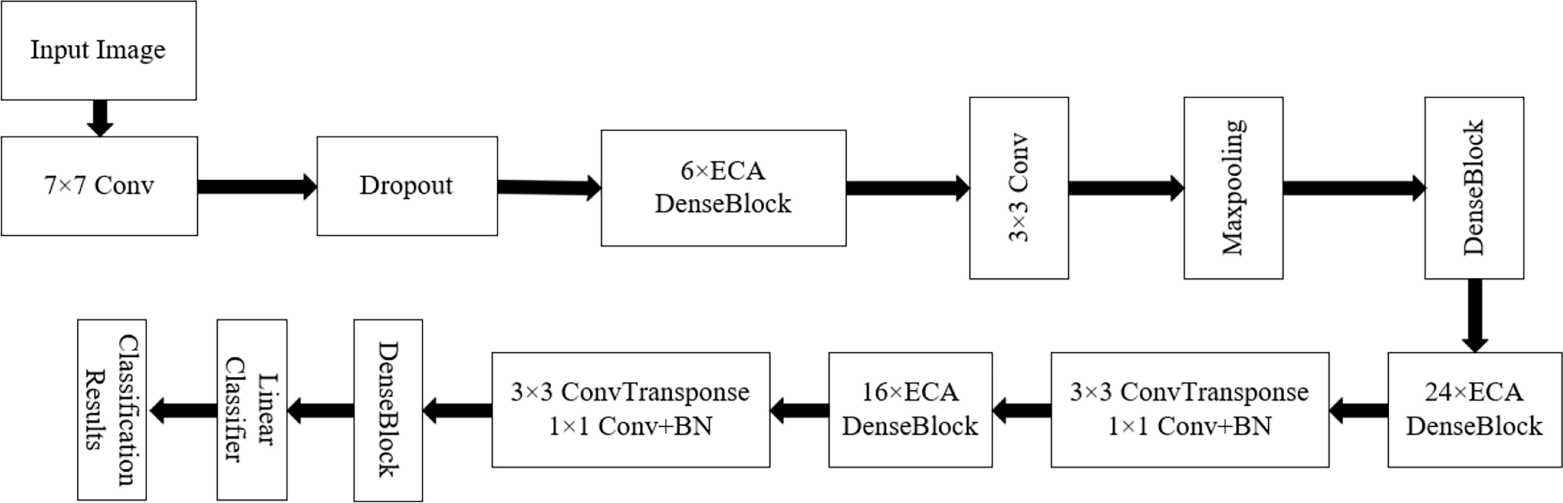
Model structure diagram.

**Figure 10:**
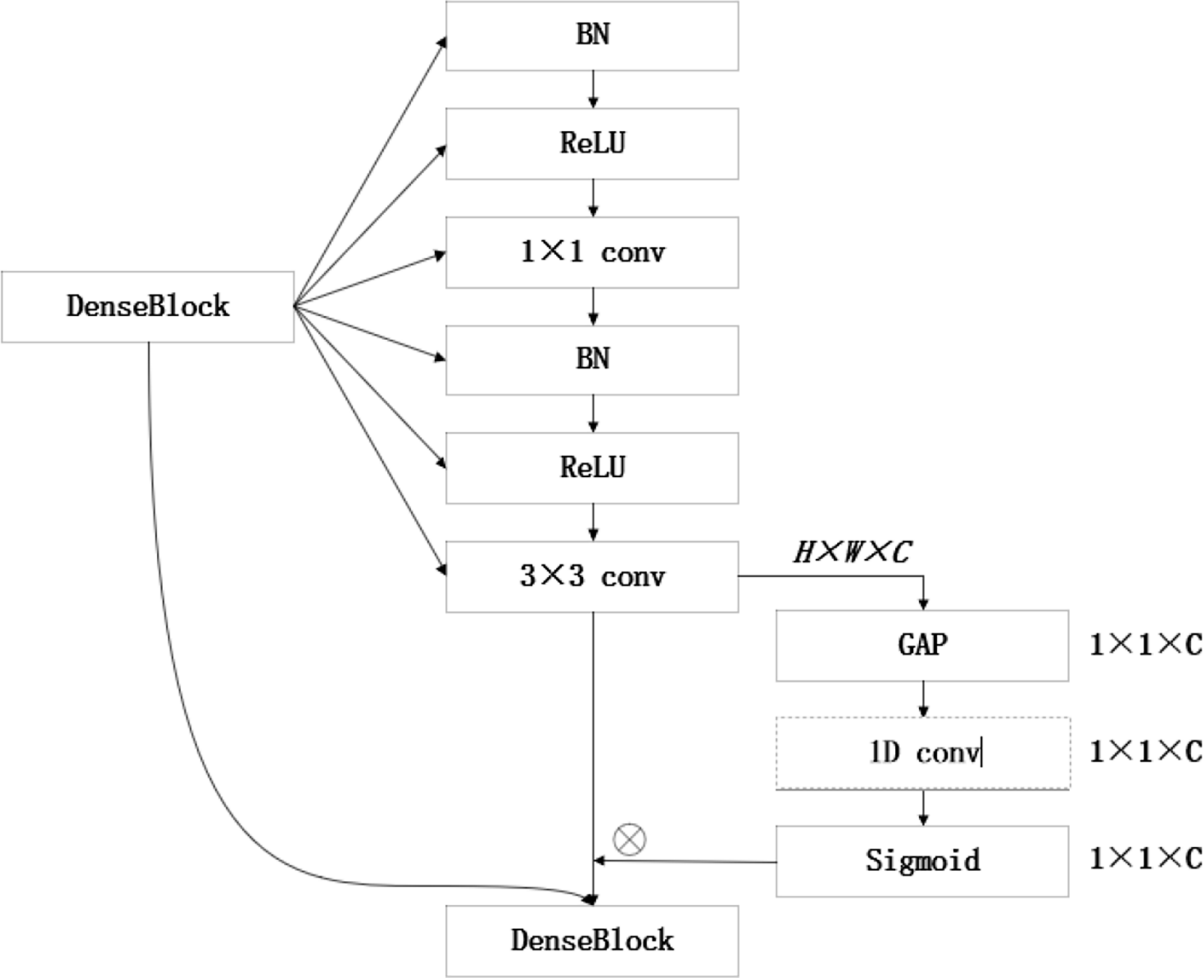
The ECA DenseBlock structure diagram.

**Figures 9**, **10** show the overall structure of the model. The input is *R*, *G*, and *B* three-channel images. First, the image passes through a 7 × 7 convolution layer. A large convolution kernel adjusts the number of channels and extracts effective information, followed by a dropout regularization layer to simulate noise, prevent overfitting, and improve the generalization ability of the model. The size of dropout is 0.5. Secondly, the ECA DenseBlock is the core part of the model. As shown in the figure, the ECA attention mechanism is added after each dense connection to increase the weight of weed features and extract more important information. The network consists of four ECA DenseBlock blocks and one DenseBlock layer. The number of improved dense connections in the ECA DenseBlock layer is 6, 12, 24, and 16, respectively, and a transition layer is connected behind each ECA DenseBlock. Among them, 3 × 3 convolution and average pooling are used to adjust the number of channels to avoid the rapid growth of feature dimensions. Combined with the symmetrical structure of U-Net encoding decoding, two 3 × 3 deconvolutions are added to further refine the target feature points in the image. After extracting features from the dense connection structure with the attention mechanism, dropout regularization is added to prevent the problem of overfitting. Finally, the class output is obtained by using global average pooling and linear classifier.

## 4 Experimental results and analysis

### 4.1 Experimental environment

The training and testing of the weed recognition network model are completed based on Keras, a deep learning framework. The hardware environment adopts the IntelXeon E5-2680 V4 CPU, and the GPU adopts the NVIDIA Ti-TAN XP graphics card and 64-GB video memory. The operating system adopts Windows 10, and Python 3.0 is used in the integrated development tool Jupiter 8.0.

### 4.2 Experimental results

In order to train the best recognition model, a series of experiments are carried out on the dataset to determine the setting of super parameters. Firstly, the number of training samples in each batch and the learning rate of the model are determined; then, the other parameters in turn are adjusted. Each test runs for 40 rounds, and one epoch represents a complete training of the model using all the data of the training set. In the experiment, the batch size is usually set to 6, which is conducive to parallel calculation and processing. The number of samples in this test is set to 16, 32, 64, and 128, respectively. After comparison, it is found that if the number of samples is set too small, the convergence of the model will be slow and too large will lead to insufficient memory and weak generalization ability of the model. Finally, the number of samples is determined as 64. The learning rate controls the update speed of network weight. Setting a reasonable learning rate can make the objective function converge to the local minimum quickly. The test selected 0.1, 0.01, 0.001, and 0.0001. The effect of model training is the best at 0.0001 (**Table 1**).

**Table 1:** Different learning rates.

Learning rate	Accuracy	Loss rate
0.1	Overfitting	
0.01	85.74%	8.74
0.001	90.03%	0.78
0.0001	97.78%	0.12

**Figure 11:**
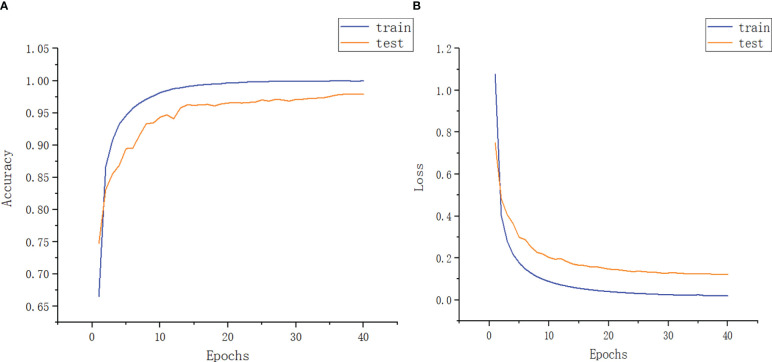
Function diagram of experimental accuracy and experimental loss rate. **(A)** Image model accuracy **(B)** Model loss rate image.

In this experiment, the Adam optimization algorithm with default parameter setting is used in model training. The algorithm is computationally efficient and requires less memory. It is suitable for solving the problems of large-scale data and parameter optimization. The experimental results are shown in **Figure 11**.

### 4.3 Analysis of experimental results

#### 4.3.1 Comparison of the different models

**Figure 12:**
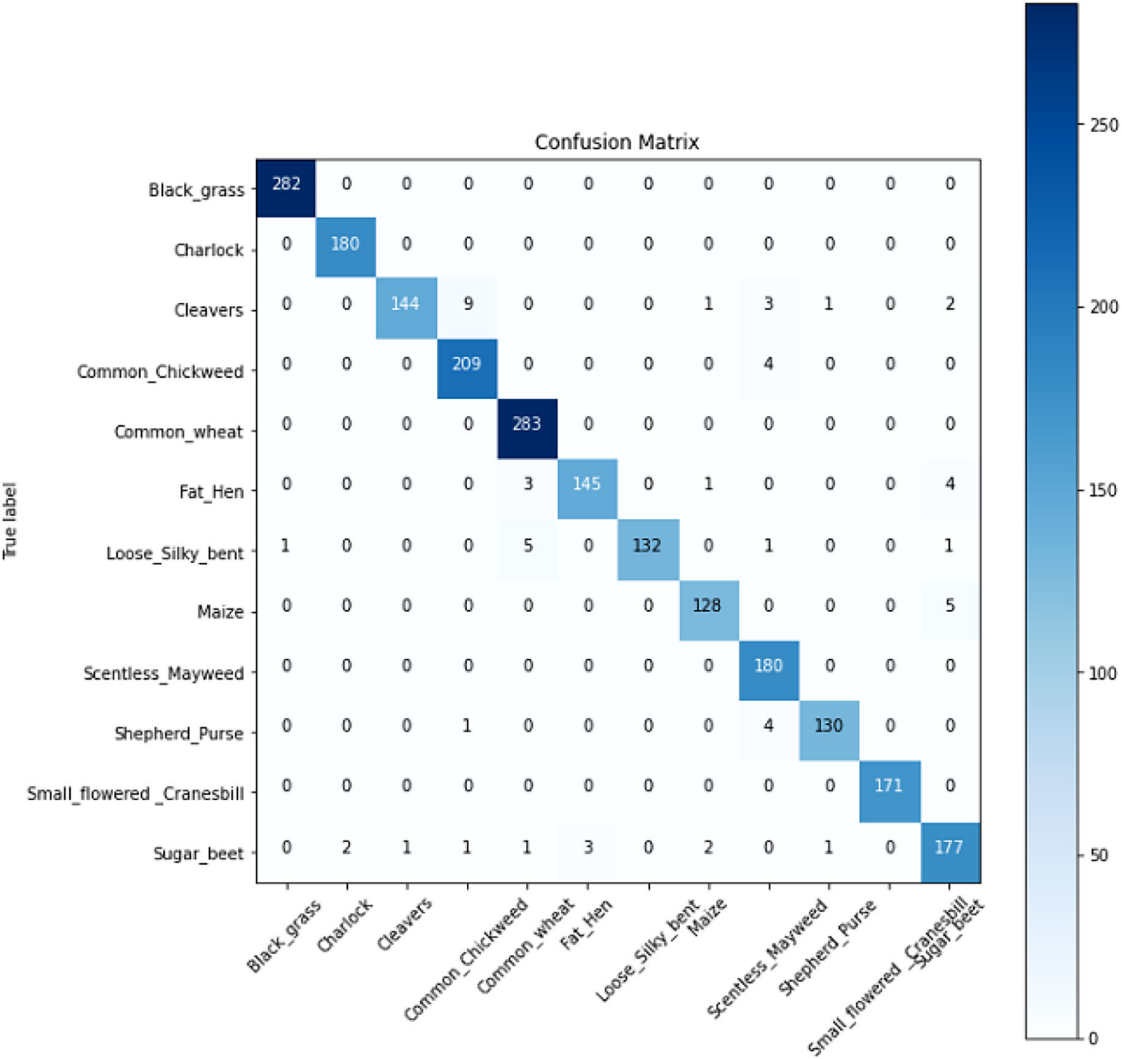
The confusion matrix of our model.

**Figure 13:**
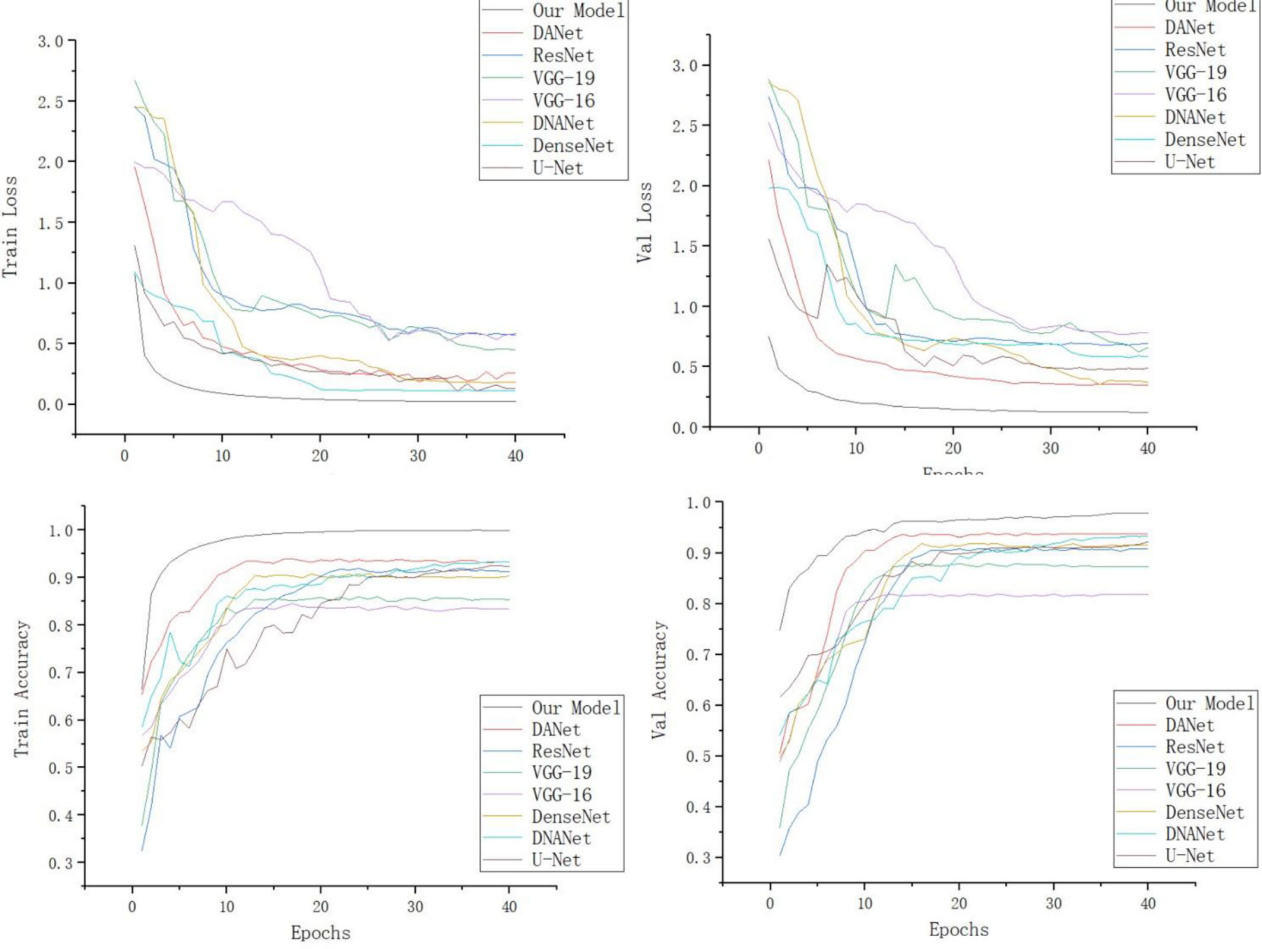
Loss rate and accuracy function images of each model.

In order to verify the validity of the model, we selected different identification models for the comparison tests under the same experimental conditions. Since VGG-16 (Simonyan and Zisserman, 2014), VGG-19 (Simonyan and Zisserman, 2014), ResNet-50 (He et al., 2016), DenseNet, DANet (Fu et al., 2020), DNANet (Ren et al., 2021), and U-Net, which are standard deep convolutional divine meridian models commonly used for image recognition in different domains, all have good recognition results, they are therefore chosen as the comparison models for this experiment. **Figure 12** shows the confusion matrix of our model. **Table 2** shows the experimental comparison results of each model. In order to more intuitively reflect the accuracy rate of our model compared with other models, the accuracy rate function diagram and loss rate function diagram of each model are shown in **Figure 13**.

**Table 2:** Experimental results for each model.

Model type	Model size (MB)	Training accuracy (%)	Testing accuracy (%)	Single image recognition time (ms)
VGG-16	800.33	83.45	81.78	153.3
VGG-19	832.45	85.34	87.23	163.1
ResNet-50	95.23	90.33	91.54	104.5
DenseNet	93.43	91.34	90.78	98.8
DANet	611.1	93.30	93.29	85.3
DNANet	169.2	92.32	92.20	88.0
U-Net	90.42	93.23	93.76	77.3
Our model	83.50	99.97	97.78	68.4

It can be seen from **Table 2** that the test accuracy of the VGG-16 model is 81.78%, which is not suitable for weed identification. The recognition accuracy of the VGG-19 model is higher than that of the VGG-16 model. This is because VGG-19 increases the performance of the model on the basis of VGG-16. The main reason is the three-layer convolution based on VGG-16. But it also increases the amount of computation and model memory. Compared with VGG, the ResNet-50 model based on different sparse structure design reduces a large number of model parameters and has a significant improvement in performance, and its test accuracy is over 90%. The U-Net model mainly strengthens the extraction of feature points because of its coding and decoding structure, so the accuracy also reaches more than 90%. The test accuracy of the DenseNet model is 93.43%, which is significantly better than that of the other models, and it has very little memory (5 MB). DANet is a dual-attention mechanism, so the extraction effect is more obvious, and its accuracy is above 90%, while DNANet is a small target extraction, which can extract the nuances of weeds, and the accuracy is also above 90%, but the memory of both models is too large and the number of parameters is more. The table also lists the detection time of this model and other depth network models for recognizing a single weed image. Each depth neural network model tests a single image for 10 times and finally takes the average test time as the test result. It can be seen from the test results that VGG-19 takes the longest time to detect a single picture, and the average detection time is 163.5 ms. The detection time of ResNet-50 and U-Net is 104.5 and 77.3 ms, respectively, while that of DANet and DNANet is 88 and 85 ms, respectively. The average time of single image detection of this model is only 68.4 ms, which is more suitable for the rapid detection of field weed images.

In this paper, the model combines DenseNet with U-Net for optimization and improvement, which effectively improves the accuracy of weed recognition. Weeds need to increase the depth of the model because they have similar feature points and similar color to corn seedlings, and need a large number of images for training the model and also bring a large amount of computation. DenseNet, through its own dense connections, reduces the memory of the model as much as possible on the basis of ensuring that the model can be trained in depth. Because the appearance similarity between weeds and corn seedlings is relatively high, we need to extract some subtle features of weeds and corn seedlings as finely as possible and use these features to classify corn seedlings and weeds. The U-Net network satisfies this requirement, and the U-shaped structure is used to encode and liberate the image, perform fine feature extraction on the enlarged image, and extract the features that cannot be recognized by the naked eye, so as to achieve the effect of the model for weed recognition.

#### 4.3.2 The effect of image preprocessing on the model

Most of the weeds in the planted land are grassy herbs that are fine and dense. In contrast, weeds have wider leaves and sparse foliage. In the image of turfgrass containing weeds, weeds are used as foreground and turfgrass as background, and the grayscale varies widely among dense turfgrasses, while weeds have wider leaves and uniform grayscale variation. Therefore, this paper uses the method of preprocessing the image after local variance, and this method is consistent with the image obtained by directly passing the original image through high-pass filtering, and finally both of them get the high-frequency part of the image. This part can be enhanced appropriately, making the image to become clearer. At the same time, in order to prevent data overfitting, a data enhancement process is needed. In order to prove the effectiveness of the algorithm, the dataset is divided into four parts and input into the model we designed, and the experimental results are shown in **Table 3**. The experimental result is the average of the results of five experiments.

**Table 3:** Results of the four datasets run in our model.

Data number	Data processing methods	Accuracy
A	No processing method	85.96
B	Background split	90.35
C	Data enhancement	91.44
D	Background split and data enhancement	97.78

In **Table 3**, the weed identification network models were trained on four datasets using different ways of processing the datasets, and the accuracy of the obtained models was compared, as can be seen from **Table 4**: the accuracy of the dataset obtained by inputting the unprocessed dataset into the model is 61.69%, while the accuracy of data B and C is significantly higher than that of A (**Table 3**), indicating that the performance of the model has been greatly improved. The accuracy of data D is the best among these cases, in which the accuracy of the un-data augmented datasets A and B is significantly different compared with that of data C and D (**Table 3**), which indicates possible model overfitting when the model is trained on the un-data augmented dataset. It is also shown that the local variance algorithm splits weeds and corn seedlings clearly, so that the deep learning model can better extract finer features of weeds and corn seedlings, remove factors that may affect the experimental results, and finally achieve the goal of improving the accuracy. In order to verify the universality of the method, which can be used in other models in the future, we input the dataset into DenseNet, VGGNet-16, VGGNet-19, ResNet-50, and U-Net for experiments, and the experimental results are shown in **Table 5**. It is proved that the method can separate complex backgrounds and be used to extract target features centrally and is not only applicable to a single model (**Table 5**).

**Table 4:** Data amplification.

Weed species	Raw data	Extended data
Black-glass	309	618
Charlock	452	904
Cleavers	335	670
Common chickweed	713	1,426
Common wheat	253	506
Fat hen	538	1,076
Loose silky-bent	762	1,524
Maize	257	514
Scentless mayweed	607	1,214
Shepherd purse	274	548
Small-flowered cranesbill	576	1,152
Sugar beet	463	926

**Table 5:** Results of different data processing runs on each model.

Model type	Data processing available	No data processing
VGG-16	83.45	62.67
VGG-19	85.34	65.22
ResNet-50	90.33	81.43
DenseNet	91.34	85.32
DANet	93.30	87.34
DNANet	92.32	89.24
U-Net	93.23	86.95
Our model	97.78	93.46

#### 4.3.3 Impact of ECA on model performance

To verify the effect of adding the attentional mechanism ECA, the experiment was also divided into sections with and without the attentional mechanism ECA. As reflected in **Table 6**, the accuracy of the model was significantly improved after the addition of the attention mechanism ECA, which is due to the fact that the addition of the ECA attention mechanism in the feature extraction process can effectively enhance the extraction of weed features in complex backgrounds and further distinguish the difference between weeds and maize seedlings. Because the data are segmented by background, some redundant information is removed, so although the attention mechanism ECA is added, it does not concentrate too much on extracting other information to ensure the model’s accuracy. In addition, the attention mechanism ECA can prevent the overfitting phenomenon that the model has good recognition ability in the training phase and poor recognition ability in the testing phase, and ensure that the network learns the correct feature information and improves the accuracy of the dataset substantially. Therefore, combining the improved model with the attention mechanism ECA ensures the accuracy of the model for weed recognition and crop.

**Table 6:** Experimental results of our model with and without ECA.

Type	Accuracy (%)	Length of training per round (s)	Single image test duration (ms)
With ECA	97.78	132	68.4
Without ECA	94.34	145	71.2

## 5 Conclusion

In order to solve the problems of low accuracy and weak generalization ability of weed species and crop identification in a crop field in a complex environment, a weed identification method based on improved DenseNet was proposed in this study. On the basis of the DenseNet network, the ECA mechanism is introduced to strengthen the extraction of weed features.

1) The average recognition accuracy of the model proposed in this paper can reach 97.78%, higher than the DANet, DNANet, VGGNet-16, VGGNet-19, ResNet-50, U-Net, and DenseNet models without improvement. Compared with the improved model, it is improved by 7.2 percentage points, which verifies the effectiveness of this model in weed identification.
2) The size of the improved DenseNet network model is 83.5 MB, and the time consumption of a single picture is 68.4 ms, which are better than the other networks and can be easily deployed to intelligent weeding equipment.
3) Data enhancement and background segmentation of the data using local variance and the super green method can obtain higher recognition rate, which can remove complex background, enhance the generalization ability of the model, and improve the robustness of the model.

The research results of this paper have implications for the identification of other crops with associated weeds, and by testing and improving existing algorithms, the generality of the model for weed identification and crop problems can be improved. In the future, models can be implanted into mobile devices for precise detection of farmland, leading to targeted weed control and improved crop production efficiency.

## Data availability statement

The original contributions presented in the study are included in the article/supplementary material. Further inquiries can be directed to the corresponding author.

## Author contributions

YM was involved in the pre-experimental investigation, the compilation of the code, the management of the experimental team, and the writing of the paper. RN was involved in running the code and in revising and writing the paper. LF was involved in writing and correcting the paper. TL was involved in the translation of the thesis. HP was involved in the correction of the paper after translation. RF was involved in the creation of the images in the paper. JL participated in writing and correcting the equations in the paper. YW was involved in the selection of the dataset in the paper. YB was involved in the pre-research of the paper. YG participated in the preliminary research and dataset screening of the paper.TH was involved in organizing the experimental data. HG was involved in reviewing, organizing, and summarizing the data related to the thesis. SL was involved in reviewing, organizing, and summarizing the data related to the thesis. YS was involved in reviewing, organizing, and summarizing the data related to the thesis. All authors contributed to the article and approved the submitted version.
